# A family caregiver perspective: rethinking recovery with phenomenology

**DOI:** 10.3389/fsoc.2025.1509351

**Published:** 2025-01-17

**Authors:** Junyu Ke

**Affiliations:** Centre for the Studies of Theory and Criticism, Western University, London, ON, Canada

**Keywords:** phenomenological experience, family caregiver, social roles, body–mind, care

## Abstract

Our phenomenal experience of the world is shaped by lived moments of the present, which not only sediment into the fabric of our current reality but also actively contribute to shaping it. We continually engage in the generative and rich making of life through this ongoing, dynamic interaction with the world. From this perspective, body–mind differences resulting from brain injury could be seen as a profound transformation of one’s phenomenal experience of the world. The lessons I have drawn from my caregiving experience with my sister who has critical brain injuries highlight the need to move away from ableist beliefs that disabilities are deficits to be corrected or rejected to a positive and generative search for the new, alternative ways of living well with shifted physio-psychological conditions. Using phenomenological perspectives, I aim to shift the understanding of “abnormality” from the binary of normal/abnormal to a broader vision of care. For family caregivers who struggle to help their loved one to return to a better state of health and life quality, the key point of participating in the recovery process is to gear into the lived experience of the care recipient and grasp a genuine understanding of their reality.

## Introduction

My sister experienced a hemorrhagic stroke in 2019 and was placed in ICU for further observation after receiving a craniotomy. Years later, she is still diagnosed as having six types of disorders, including movement and cognitive disorder. Though with constant reminding she seems able to acknowledge her roles as mother, wife, sister, and daughter, she does so without any sense of actual commitment to them. Her previous understanding of herself and the world, as well as the relation between the two, seems to have been disrupted by the change in her bodily condition. Her ability to hold on to her previous social roles and fulfil the norms expected of her has also been largely hampered due to brain injury. She cannot connect to the world and the people around her in the same way. The disruption manifests in her impeded capacity to anchor meanings from her past to inform her present. From a phenomenological perspective, she has undergone a profound change in her way of being and her ways of experiencing the world. The way she situates herself in relation to others has changed.

More often than not, my sister is addressed as a patient even when she returns home. Although her surgery was performed and the wound was closed, the disease remains. As Canguilhem (1978) would agree, this judgment has more to do with self-appraisal and the dominant ideas of society than merely physician’s opinions. Disability shapes an individual’s relationship with their environment and the broader socio-cultural context in which they live, situating people in unique worlds that, while different, remain part of the larger shared world with able-bodied individuals. This interconnectedness highlights the intersection of personal and social experiences of disability, where both disabled and able-bodied identities are shaped and intertwined. Writing about disability, therefore, is not merely an act of storytelling but an ethical and communal responsibility. It creates space to amplify marginalized voices and critique the social systems that perpetuate exclusion and oppression (Price, 2011; Siebers, 2008). Through this lens, disability is not just a condition but a politicized identity that challenges historical and cultural meanings ascribed to it (Garland-Thomson, 1997; Kafer, 2013). Writing about disability, particularly from the perspective of a caregiver, becomes an ethical engagement of transcending oppressive binaries.

In this light, instead of seeing my sister’s disconnection/nonconformity to her previous social role as a failure, I will reassess her reality with a broader vision provided by phenomenology, in which the understanding of “abnormality” stemming from binary thinking is interrogated. The interrogation then opens up a search for new, alternative way for the person and their community to live. Drawing upon Merleau-Ponty’s (2012) work, *Phenomenology of Perception*, I will explore the phenomenological constitution of experience, meaning the way experiences are structured and informed by phenomenological insights. One particular focus is on the latent content of experience that encompasses the sedimented past and the spontaneous embodied interaction with the present. This perspective will show how social and cultural specificity, as well as bodily conditions, shape one’s phenomenal experience of the world. Ultimately, caregivers should engage in a search for “conditions of possibilities” (Messas et al., 2018) that would genuinely support the care recipients in their unique circumstance and lived reality, rather than attempting to suppress or homogenize their experience. By reflecting on my subjective experience of encountering my sister as a newly disabled body–mind subject through a phenomenological perspective, I intend to reconcile my anxiety as a family caregiver and her shifted psycho-physiological state.

## The phenomenological constitution of experience

In Merleau-Ponty, there is a central focus on the body as the primary means through which we experience and engage with the world. The body as an anonymous, shared cultural body arises from the deep intertwining of individual embodiment and cultural context. It is not just a passive object but an active perceiving subject that intends, desires and acts in the world. A newly disabled body, as my sister is currently living with, inevitably constrains the subject’s bodily autonomy to engage with the surrounding world as they did in the past, thereby placing them in an unfamiliar territory that requires a reunification of movements, senses and ideas. This process entails a transformation in the perception of the *Other-for-me*, in Sartre’s (1943, p. 343) words. Here, the body is not only a personal experience but also an object in the social realm as seen and experienced from the perspective of the Other.

The initial step, acceptance rather than resistance, is of utmost importance, as I learnt through my caregiving experience with my sister. We are often perceived in roles dictated by normative expectations, which prescribe certain traits for how people should behave within those roles. In the urban China context, especially in a metropolis like Shenzhen where my sister lives, there is an intensifying demand on mothers to become more knowledgeable and skillful in providing care to their families, particularly in regard to child development and parenting (Zhang et al., 2023; Wang et al., 2024). For example, engaging school-age children in various extracurricular activities such as English and piano classes is considered an essential aspect of good motherhood (Meng, 2020). As a mother, my sister’s ability fulfill these expectations was not just a personal achievement but a marker of social belonging. After her brain injury, however, her inability to maintain these traits caused us, as her caregivers, discomfort and a compulsion to guide her back on track. This discomfort stemmed from an ingrained fear that her divergence from these norms would render her marginalized in the eyes of society, which tends to judge individuals, especially mothers, against rigid standards of productivity and care.

My sister underwent early rehabilitation involving cognitive, linguistic, motor, and swallowing therapies, supplemented by acupuncture and electrotherapy. In this process, we often felt frustrated by the lack of steady progress, especially when her emotional fluctuations disrupted treatments. Her significant muscle tension, impeding limb recovery, felt like obstacles rather than integral parts of her healing, leading to frequent misunderstandings and frustrations. However, a reflective approach revealed that we had prioritized a return to “normalcy” over my sister’s subjective and nuanced experience, and that her differences, brought out by her new reality, were not adequately considered or accommodated in the rehabilitation setting as we prioritized conventional measurable outcomes over exploring a unique recovery path tailored to her changed conditions. Moving forward, it is crucial to understand the implications of being seen as “deviant” from conformist expectations of the “normal,” and how that affects our choices and goals in assisting our disabled loved ones. Embracing a broader perspective on disability and differences can be both empowering and challenging.

## The norms of body

As my sister becomes incapable of fulfilling any of her previous social roles in her new body–mind conditions, she is no longer seen as a productive member of the society, and thus becomes susceptible to being devalued, objectified, or rejected as a burden. Dosanjh et al. (2021, p. 336) also observed divergent copings with long-term FMD symptoms; some people continued to suffer stress, shame, and anxiety, or mourned their loss, while others “were able to value themselves in new, albeit more limited, roles” after a period of adjustment. Although a disabled body can have severe consequences on individual wellbeing and health (e.g., dissolution of instrumental social relationships and bonds that leads to social isolation), it is not inherently harmful to develop and maintain a meaningful life and identity. Individuals are not passive recipients subject to the reward-punishment mechanisms of social systems. The body of my sister is subject to value judgment of the society.

However, the dependency of the subject’s meaning-making on social norms is only threatening if we predicate the notion of subjectivity on the ideal of a self-possessed sovereign subject (Oliver, 2001). By embracing an interrelational perspective of subjectivity and acknowledging the profound effect of social relationships on one’s sense of self and agency, “dependence is seen as the force of life, as the very possibility of change,” as opposing to a one-way drive to violence and death (68). Such a view of dependency is upheld with Levinasian ethics, that relations to the other entails ethical responsibilities, where justice emerges. In her later work, Oliver (2004, p. 199) is more explicit, stating that “ethics is the acknowledgment that we are by virtue of response from others and by virtue of response from meaning through which we become beings who mean. Subjectivity, then, is inherently ethical. We are subjects or subjectivity only through our relations with others, and ultimately with otherness.” Although our subjective experiences and social relationships are predominantly shaped by the overarching social system that imposes distinctions and exclusions, a change in one’s social identity—such as becoming disabled—offers an opportunity to reevaluate how we interpret and respond to social interactions. In my sister’s case, her psychic space is to be reimagined within the confines of her shifted physical circumstances and reduced social connections. For the family caregivers, we become aware of our unconscious hostility to difference or deviance from the unquestioned norms, as we witness the challenges and injustices faced by our loved ones who have become disabled: we were once shut out of the elevator because moving my sister in her wheelchair made us too slow for impatient passengers; my five-year-old niece, my sister’s second child, cried out of boredom at home because her mother was always sedentary and inert; and my mother kept mourning why my sister still had not regained her “consciousness,” in the sense of being aware of who she used to be. The individuality within the new conditions is ignored. And this is a critical point concerning why overcoming does not work, why diagnosis obscures more than it reveals, and why cure tends to essentialize the reality rather than provide a holistic approach to who we are and what we need in order to lead meaningful lives with the changed body–mind conditions. Recognizing the newfound individuality asks for not only individual perspective shifts but also broader changes in social perception and attitudes that maintain the power of the ableist framework.

## The new understanding

Once I embraced a phenomenological understanding of how to approach my sister’s changes, I began to recognize and give meaning to her potential within these new circumstances. I realized that she has other possibilities beyond those defined by societal norms and expectations, which are no less significant for her and the community than her previous ones. For example, while she may not exhibit a proactive preference for exploring her environment, she shows interest when prompted, such as when asked if she wants to visit the kitchen. Once in the kitchen, she expresses curiosity and finds enjoyment in the new surroundings, particularly when guided to observe specific elements like plants. This indicates that with intentional guidance, she can form temporary connections with her environment. Additionally, as her mobility improved, she exhibited some agency, demonstrating that her potential for engagement and interaction with her surroundings can be activated and nurtured. As Oliver stresses, we are not only responsible for our fears, desires, and emotional expressions, but also for their effects on others, “we are responsible for the other’s response.” (199) This acknowledgement of the ethical responsibilities to others is the departing point where we “forgive,” or in other words, embrace the uniqueness and individuality of others within the fabric of social coherence. My role as a family caregiver has afforded me an opportunity to conduct a “self-critical hermeneutics” that involves examining and questioning my own ableist-centered perspectives regarding my sister’s changes. Reflecting on my previous emotions and desires, mostly anxiety and frustrations regarding the slow progress of my sister in reconstructing her mobile and cognitive capacities, I become more accepting of her conditions and changed my approach to meet within those limitations. Now, I choose to disregard what she cannot do and focus on what she can do. Through this shift in perspective, understanding and supporting my sister’s rehabilitation is no longer filled with a sense of urgency to bring her back to “normal,” especially as this direction is not only futile but counterproductive given the dramatic physical and mental alternations.

Ableism, according to Campbell (2009, p. 5), is a “conceptual tool… a chief feature of an ableist viewpoint is a belief that impairment or disability (irrespective of ‘type’) is inherently negative, and should the opportunity present itself, be ameliorated, cured, or indeed eliminated.” Failure to meet the normative standards is seen as “deviance” from what is generally accepted as normal or the norm. Clare (2017, p. 8) warns, “[that] disabled people can only succeed by overcoming disability is an ableist cliché.” The fear that my sister will be devalued and excluded from society due to her disabilities dominated my approach to her new reality; I failed to understand her needs and provide her with adequate support. Unable to access my sister’s world, I imposed goals on her, hoping we could “step-by-step,” “gradually and quantitatively,” “effectively,” overcome her deficits and aid her once again in becoming able-bodied. My demands to correct the perceived pity and wrongness of my sister’s disability have been conditioned by my able-bodied perspective and the internalized ableist norms that our society perpetuates. “The common narrative that we endorse is ‘overcoming’, which is filled with “unjust ability expectations determining how bodies should be in the very recesses of how they are” (Reynolds, 2019, p. 5). The concept reduces disabilities to merely functional defects in the physical and/or mental realm and disregards the vitality of differences of body–mind manifestations, assuming that the located problems can be fixed, contained, or eradicated.

## Discussion

My reflection on my sister’s actions, speech, and affections points to the need for unlearning the learned, unknowing the known, and undoing thoughts structured by habit, custom, rules, and other social constructs. In my personal experience, the relationship between disabled people and caregivers is often characterized by independence and involves power imbalances leading to mistreatment and exploitation of the person receiving care, which can occur across settings, from home-based caregiving arrangements to hospitals. However, in light of evolving scholarship, this dynamic has been critically reimagined through more relational and reciprocal models of care. Bellacasa (2017) compels us to consider “the meanings of care as a noninnocent but necessary ethos of always situated implications” (p. 24), emphasizing care as a way of knowing and thinking connectedly—about humans, nonhumans, and the systems that entangle us. Similarly, Nishida (2022) advocates for moving beyond a dichotomized understanding of care to embrace it as a situated practice, deeply attuned to the particularities and complexities of another’s existence. This reconceptualization shifts care from being a set of prescribed actions or outcomes to an empathetic and adaptive practice that honors the changed body–mind conditions of the care recipient, as in my sister’s case.

When I set aside goals based on the dominant norms of our ableist society and stop assuming that my sister can be restored to her former self, the recovery process transforms. It becomes a journey searching for day-to-day approaches attuned to her shifted subjective experience of the world. I shift my focus from major milestones to celebrating small achievements, such as her managing to do one or two more leg lifts than in the previous session. Whenever she made even minor progress, I cheered her on, which clearly brought her joy. This perspective not only aids my sister in her functional recovery but also encourages her to discover new ways of being and interacting with the world. The tension between homogenizing for standards and accommodating the individual can thus be eased with an extensive vision of returning to health. As Clare (2017, pp. 14–15) points out, the essence of restoration is to understand and align knowledge, experience and expectation with the unique rhythms of the new conditions; it is “a fluid, responsive process…requires digging into the past, stretching toward the future, working hard in the present. And the end results rarely, if ever, match the original state.” For family caregivers, engaging deeply with the care recipient’s lived experience is essential to help them find meaningful ways to thrive within their physical and mental limitations. My own caregiving journey has provided a valuable opportunity to reflect on how conventional understandings of disability, cast in the ableist logic, can shape our approaches and attitudes. This reflection entails broader implications for both clinical and social contexts. It underscores the importance of fostering not only individualized but also deeply empathetic healthcare philosophies and practices that attune to the unique contexts of each patient. Socially, it challenges and encourages a paradigm shift in public perceptions, seeking to build greater inclusivity and a more compassionate society where differences are not just accommodated but valued.

## Conclusion

My conceptual reflection on my caregiving experience of my sister starts from my pondering on what it means for her to be disconnected from her previous social roles. Fulfilling one’s social roles is essential for one to be properly accepted and positioned in social relations, which also delimits personal attitudes and choices. Body–mind differences resulting from brain injury could be seen as a profound transformation of one’s phenomenal experience of the world. The lessons I have drawn from my caregiving experience highlight the need to move away from ableist beliefs that disabilities are deficits to be corrected or rejected to a positive and generative search for the ideal ways of living well with the shifted physio-mental conditions. For family caregivers who struggle to help their loved one to return to a better state of health and life quality, the key point of participating in the recovery process is to gear into the lived experience of the patient and to grasp a genuine understanding of their reality. In this way, both the family caregiver and the patient are better off finding coexistence, if not consensus, of values and beliefs in the diverse forms of human bodily-being.

## Data Availability

The original contributions presented in the study are included in the article/supplementary material, further inquiries can be directed to the corresponding author.
